# Proteomic Approaches for Studying Alcoholism and Alcohol-Induced Organ Damage

**Published:** 2008

**Authors:** Susanne Hiller-Sturmhöfel, Josip Sobin, R. Dayne Mayfield

**Keywords:** Chronic alcohol consumption, chronic alcohol effect, proteomics, genomics, proteins, protein expression, gene expression, proteomic analyses, genomic analyses, protein–protein interactions, shotgun proteomic studies, biological markers

## Abstract

Proteomics research is concerned with the analysis of all proteins found in an organism, tissue, cell type, or cellular structure. The shotgun proteomic approach, which involves two-dimensional gel electrophoresis or liquid chromatography combined with mass spectrometry (MS), is used to identify novel proteins affected by alcohol. More targeted analyses study protein–protein interactions using such techniques as the yeast two-hybrid system, affinity chromatography, or immunoprecipitation. Finally, proteomic strategies can be combined with genomic research findings using computer analyses (i.e., in silico). All of these approaches have been used in the alcohol field. These studies have identified proteins in various brain regions whose expression is affected by alcohol. Other investigators have used proteomic approaches to identify proteins that could serve as potential biomarkers of alcohol use. Finally, interaction proteomic analyses have begun to identify proteins involved in several nerve signaling networks in the brain, which then can serve as targets for further studies on alcohol’s effects. Future proteomic studies likely will shed more light on the mechanisms underlying alcohol’s actions on the body.

One of the central physiologic processes occurring in any organism is the conversion of the genetic information encoded in the DNA into proteins. This process, also known as gene expression, consists of two consecutive sets of chemical reactions:

- Transcription, in which an intermediary molecule called messenger RNA (mRNA) is generated based on the genetic information of the DNA; and
- Translation, during which the mRNA serves as a blueprint based on which proteins are synthesized from their building blocks, the amino acids.

These proteins then form cells and their structural components or act as enzymes that help generate and metabolize almost all other molecules that are needed for the organism to function. The coordinated activation of specific genes in specific cells—and the resulting production of the corresponding proteins—at specific times during development or under specific environmental conditions allow the body to grow, to maintain its activity levels, and to adapt to changes in its environment. Therefore, researchers can study the normal functions of the body as well as its responses to diseases or other abnormal conditions (e.g., acute or chronic exposure to alcohol) at both the gene and protein level.

The field of proteomics—that is, the large-scale analysis of all proteins encoded by the DNA as well as of protein complexes composed of multiple interacting proteins—has grown tremendously in the last 3 to 6 years (Husi and Grant 2001; Pandey and Mann 2000; Wolters et al. 2001). Particularly, the development of techniques to study protein complexes and identify their components has led to important advances because many proteins are parts of networks of interacting proteins that may affect the functions of several organs and/or play a role in diverse disease states. For analyses of these networks, proteomic approaches provide an obvious benefit over traditional techniques that only allow examination of single proteins and their individual functions.

Proteomic studies have been used in a wide range of research, including the alcohol field. Such studies have the potential to greatly enhance our understanding of the effects of chronic alcohol abuse. For example, chronic alcohol abuse produces persistent changes in brain function that result in tolerance, physical dependence, craving, and other behavioral changes. These changes in brain function likely result from alterations in gene and protein expression that cause the cells to adapt to chronic alcohol exposure (Anni and Israel 2002; Nestler 2000). Identification of selective changes may lead to the development of new therapeutic approaches, as has been demonstrated in other areas of research, most notably in cancer research, where analyses of changes in gene and protein expression already have led to improved pharmacotherapies (Okutsu et al. 2002; Taxman et al. 2003; Zembutsu et al. 2002).

Compared with the study of the entirety of all genes of an organism (i.e., the genome), studies of the collection of all proteins of an organism (i.e., the proteome) are much more challenging for several reasons. First, the proteome is much larger than the genome. Whereas human cells contain an estimated 30,000 genes, the number of proteins produced has been estimated to approach 1 million (Pennington et al. 2005). Several mechanisms contribute to this diversity in proteins. For example, the genetic information contained in one gene may result in the production of several proteins through a process occurring after transcription called differential gene splicing^1^ that generates a range of mRNA molecules to be translated into different proteins. Moreover, after proteins have been synthesized during translation, they can be modified by the addition of various chemical groups. These so-called posttranslational modifications (PTMs) can make a protein more or less active. However, only few PTMs can be predicted based on the DNA sequence of the encoding gene. This makes identification of specific proteins and their PTMs much more daunting.

The second challenge associated with proteomic analyses is the huge variation in protein expression levels. When conducting genomic analyses, researchers know that all cells in an organism contain the same set of genes. Although some genes are present in more than one copy per cell, the variation in copy number is relatively small. In contrast, the level of protein expression is vastly more variable (Anni and Israel 2002). Each cell expresses only a certain subset of its genes at any given moment, depending on specific cell type, developmental stage, and environmental conditions. Furthermore, the same amount of protein is not made from each gene. Thus, for some genes only one or a few copies of the corresponding proteins may be produced, whereas for other genes many copies of the proteins may be synthesized. Finally, some proteins are produced only in certain cell types and are entirely absent in others.

A third problem is that many proteins, especially those with regulatory functions, may be present only in minute amounts. Consequently, they are difficult to detect among all the other proteins expressed at the same time in the same cells or tissues, which may be present in much greater amounts. For example, blood or other bodily fluids contain large amounts of the protein albumin and other proteins that in most cases are not relevant for the question under investigation.

The development of new technologies has enabled researchers to overcome at least some of these challenges. Despite their difficulties, genomic and proteomic approaches offer distinct advantages over “traditional” molecular strategies when investigating such complex issues as the search for genes and proteins that are affected by alcohol or that mediate alcohol’s effects. Thus, genomic and proteomic technologies allow researchers to examine large numbers of potential target molecules (e.g., proteins) simultaneously and in an unbiased fashion, without having to make prior assumptions about which molecules might be involved. In addition, both genomics and proteomics have matured rapidly over the past few years, and important advances in bioinformatics will undoubtedly improve researchers’ ability to interpret the large amounts of accumulating data and identify common underlying themes. This then may lead to the identification of alcohol-sensitive genes and proteins and increase the understanding of drug mechanisms of action.

Based on methodological considerations, proteomics research can be classified into three major areas (Kasinathan et al. 2004):

- Structural proteomics, which analyzes the physical structure of proteins, using such techniques as X-ray crystallography and nuclear magnetic resonance (NMR) spectroscopy;
- Expression proteomics, which compares patterns of protein expression levels under different conditions (e.g., in alcoholics and nonalcoholics) by separating proteins in an extract and subsequently quantifying and identifying differences between the two conditions; and
- Functional proteomics, which explores protein activities and the interactions of proteins with each other and with other components of the body using a range of diverse assays.

This article focuses on the latter two aspects of proteomics. After summarizing some general considerations for conducting proteomic studies, the article presents some of the most commonly used techniques used for these analyses. It then reviews the findings of recent studies in the alcohol field that have used diverse proteomic strategies and explores future perspectives of this research area.

## How Can the Proteome Be Studied?

### What Should Be Studied?

As mentioned earlier, the protein expression in a given cell depends on such factors as cell type, developmental stage, and environmental conditions. Accordingly, for multicellular organisms, analyzing the proteome of the entire organism at once is neither feasible nor desirable because it provides no information on the distribution or function of individual proteins. Researchers, therefore, typically focus on subproteomes—for example, the proteins found in a specific organ, tissue, or cell type of interest under certain conditions. Thus, subproteomes relevant to alcohol research could include the proteome of the entire liver or of a specific type of liver cells, of certain brain regions known to be affected by alcohol, or of certain types of brain cells (e.g., brain cells that communicate via a specific messenger molecule [i.e., neurotransmitter] such as dopamine). More purified tissue preparations allow the study of certain cellular structures that are considered sites of alcohol action and whose proteome can be separated from that of the rest of the cell. Such alcohol-related cell structures include the small bodies in the cell where certain substances are metabolized (i.e., microsomes), the cell’s “energy factories” (i.e., mitochondria), or the parts of a nerve cell (i.e., neuron) from which nerve signals are transmitted to neighboring neurons (i.e., the synapses).

The proteome chosen and the methodological approach selected depend on the specific question to be asked and the extent of preexisting knowledge. As mentioned previously, an advantage of proteomic analyses is that they can be conducted without bias and without any prior knowledge or assumptions of which proteins will be identified or studied. For example, one can attempt to separate and identify all liver proteins to determine the liver proteome. One also can more specifically examine the liver proteome in the absence and presence of alcohol in order to identify proteins whose abundance is influenced by alcohol. This so-called shotgun approach, which requires no knowledge of any specific liver proteins, to date has been most commonly used in alcohol research.

Because almost every physiological process requires interaction between different proteins, other proteomic analyses focus on identifying proteins that interact to form large functional complexes or even networks of proteins involved in regulating one specific process. This approach also is known as interaction proteomics. For example, one can try to identify all proteins involved in transmission of dopamine-mediated nerve signals in the brain that are affected by alcohol.

Finally, the results of proteomic analyses can be combined with those of genomic analyses to yield a better understanding of how alcohol or any other environmental factor impacts various steps in the conversion of the genetic information into protein. These analyses combine findings obtained at the DNA, mRNA, and protein levels.

### Commonly Used Strategies in Shotgun Proteomics

The basic concept of shotgun proteomic analyses is to generate extracts containing proteins from the desired tissues, cells, or cell components; to separate individual proteins using a process called chromatography; and then to study and identify individual proteins using techniques such as mass spectrometry (MS). Although this sounds straightforward, the challenges become obvious if one considers that an extract easily can contain several thousand proteins at a wide range of concentrations. Only through the development of automated processes has this become possible.

Some commonly used techniques for separation and identification of proteins in shotgun proteomic studies are two-dimensional gel electrophoresis (2-DE), liquid chromatography (LC), and various types of MS.

#### 2-DE

**Figure 1:**
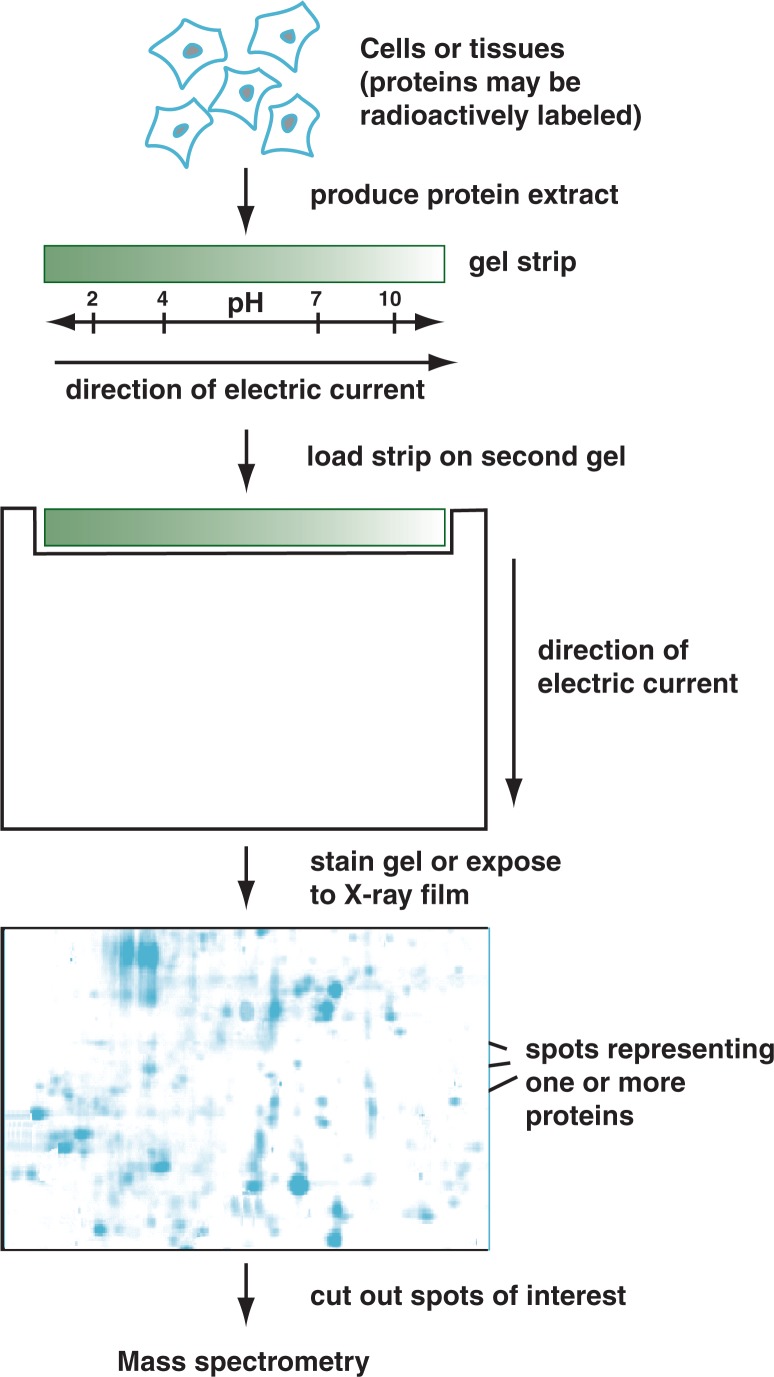
The principle of two-dimensional gel electrophoresis. Protein extracts obtained from cells or tissues first are loaded on a thin gel strip and under the influence of an electric current separated according to their electric charge (isoelectric point). The gel strip then is loaded onto another gel and exposed to a second electric current flowing in a direction perpendicular to the first one. Under these conditions, the proteins migrate from the initial gel strip into the second gel, with the distance traveled depending on the mass of the proteins. With this approach, the potentially thousands of different proteins in an extract can be separated into individual spots that can then be visualized and cut from the gel so that the proteins can be extracted for further analysis.

2-DE is used to separate the proteins in an extract based on their electric charge (i.e., isoelectric point) and on their mass (i.e., molecular weight). For this technique, protein extracts typically are first applied to thin gel strips with a pH gradient and exposed to an electric current. Under the influence of this current, the proteins migrate through the gel strip, with the distance and direction traveled depending on the electric charge of the proteins. After some chemical processing, the gel strips then are loaded on a similar type of gel and exposed to a second electric current flowing in a direction perpendicular to the first one. Under these conditions, the proteins migrate from the initial gel strip into the second gel, with the distance traveled depending on the mass of the proteins (see figure 1).

With this strategy, potentially thousands of different proteins in an extract can be separated into individual spots with characteristic coordinates on the second gel. Even proteins that differ only in their PTMs (e.g., the number of phosphate groups added per protein molecule) can often be distinguished (Abul-Husn and Devi 2006). Protein spots can then be visualized either by staining the gel with certain dyes or by labeling the proteins with radioactive groups prior to loading them on the first gel strip. Subsequently, individual spots can be cut from the gel and the proteins extracted for further analysis.

Although this technique has been one of the mainstays of proteomic analyses to date, it does have some limitations. For example, even the best gels can only separate about 1,000 proteins, and many low-abundance proteins are below the level of detection (Pandey and Mann 2000). Also, individual spots can contain two or more proteins that differ only minimally in their isoelectric point and mass and therefore are not separated. The presence of more than one protein, however, can confound the results of subsequent analyses. Nevertheless, 2-DE remains a powerful tool for studying the proteome.

#### LC

LC is a technique related to 2-DE, in which proteins are separated not on a gel but in a thin column filled with a solid material (i.e., the stationary phase) through which a buffer solution (i.e., the liquid phase) containing the protein extract is passed. Depending on specific protein characteristics, such as the size, electrical charge, and other properties, each protein is held back by the solid material for a specific amount of time. Thus, proteins that interact more strongly with the solid phase will remain in the column longer than proteins that interact less strongly with the solid phase. One then can collect consecutive drops of the buffer (i.e., buffer fractions) as they leave the column, with each fraction containing one or more proteins. These can then be analyzed further using, for example, the MS approaches described below. The number of chromatography runs performed and the specific chromatography conditions determine how well the proteins are separated and how many proteins remain in each fraction.

Several modifications of this general strategy have been introduced in recent years that enhance the effectiveness of the separation and/or facilitate subsequent identification of the separated proteins. For example, in an approach known as multidimensional protein identification technology (MudPIT), researchers treat protein extracts with an enzyme called trypsin before subjecting them to LC (Abul-Husn and Devi 2006). Trypsin is a protease, an enzyme that cleaves proteins into smaller pieces (i.e., peptides) at specific sites in the protein. Thus, each protein yields a specific set of peptides after trypsin treatment. These peptides then can be analyzed using multiple LC runs or other approaches. This strategy has several advantages over traditional LC and 2-DE. For example, it allows identification not only of soluble proteins but also of proteins bound to the cell membrane, which cannot be analyzed using 2-DE. Furthermore, the MudPIT approach is suitable for the identification of PTMs (Abul-Husn and Devi 2006).

#### MS

MS is an analytical technique used to identify the protein(s) isolated from a gel spot or LC fraction. The isolated proteins first are treated with trypsin or another protease. Then, the masses of the resulting peptides are analyzed by one of the MS procedures described below. Next, computers generate a list of the masses of all measured peptides. This list is compared with databases of known and predicted proteins and of the predicted peptides if these proteins also were treated with trypsin. If a certain number of the peptides from the unknown protein correspond to the peptides predicted for a known protein in a database, then the unknown protein has been identified with high likelihood and can be studied further based on this assumption. If no match to the peptides from the unknown protein is found in the databases, additional analyses must be conducted to identify the unknown protein. MS methods are very sensitive, highly accurate, and can identify proteins present in minute amounts.

Specific MS procedures used in proteomic analyses include the following:

- ***Matrix-assisted laser desorption/time-of-flight MS***. For matrix-assisted laser desorption/time-of-flight (MALDI-TOF) MS, the peptide mixture is combined with a matrix material and exposed to a laser beam to generate electrically charged (i.e., ionized) peptides. These ionized peptides then are injected into a detector tube, in which they travel from one end to the other, with the travel time determined by the mass of the peptides. By measuring the travel time of each peptide, the detector can generate a list of the masses of all peptides that can be compared with databases of known proteins and their trypsin-generated peptides (Mann et al. 2001). This also is known as peptide mass mapping. This approach is limited, however, by the fact that it works only if the protein under investigation already is found in existing protein databases.
- ***Electron-spray ionization combined with tandem MS.*** Electron-spray ionization (ESI) combined with tandem MS is used for peptide mixtures that cannot be ionized efficiently using the MALDI technique. For ESI, the peptides generated by trypsin treatment are ionized in a solution. These parent ions then are sprayed into a tandem mass spectrometer. This device can separate peptides in a mixture from each other, isolate one peptide at a time, and then break this peptide apart even further into daughter peptides that are analyzed by MS (Pandey and Mann 2000). Although this approach technically is more complex than MALDI-TOF, especially as more MS steps are combined, it has the advantage that it generates much more specific information on the exact sequence of the building blocks (i.e., amino acids) making up the protein. These data can be compared not only against protein databases but also against databases of short DNA pieces for which the corresponding peptides can be predicted (Pandey and Mann 2000).

#### Quantitative Proteomic Strategies

Several technologies have been developed to identify, quantify, and compare proteins in two or more complex samples. Typically, these techniques utilize stable radioactive molecules—known as isotope-coded affinity tagging (ICAT) reagents—to differentially label the proteins in the samples. Thus, proteins in extract A (e.g., liver cells from a nonalcoholic person) are labeled using reagent X and proteins in extract B (e.g., liver cells from an alcoholic person) are labeled using reagent Y. Then, the two extracts are mixed and subjected to 2-DE or LC and/or MS. With each of these approaches, one can distinguish two versions of each protein or peptide, one with the X tag and one with the Y tag, for which the relative abundance can be determined. This allows identification of those proteins or peptides that differ in abundance between the two extracts. These molecules can then be analyzed further for identification.

A similar, recently developed technique uses a different type of labeling reagent known as isobaric tags for relative and absolute quantitation (iTRAQ), which allow simultaneous analysis of up to four protein extracts using tandem MS (Aggarwal et al. 2006). This technology has great potential to improve the sensitivity and quality of MS analysis of the proteome. Its accuracy recently has been confirmed using defined protein mixtures and extracts from cells grown under controlled conditions (Unwin et al. 2005).

### Interaction Proteomics

Interaction proteomics approaches are critical, given that for most physiological processes many proteins act in concert, often directly interacting with each other. For example, proteins involved in the transmission of nerve signals from one neuron to the other have been shown to form large complexes of interacting proteins with diverse functions, such as synapse assembly and signal transmission (Garner et al. 2000). To understand the effects of alcohol and other modulators on brain function and nerve signal transmission, it is therefore crucial to characterize and identify the complex protein–protein interactions that exist in the central nervous system.

For interaction analyses, at least one component of such a protein complex must be known or at least suspected. This protein can be used as a “bait” to trap and analyze other proteins with which it interacts. Three commonly used strategies for these types of analysis include the yeast two-hybrid screens, affinity chromatography, and immunoprecipitation approaches.

#### Yeast Two-Hybrid Screens

The classical method for identifying protein–protein interactions is the yeast two-hybrid method. It is based on the observation that certain proteins regulating gene expression (i.e., transcription factors) in yeast and other higher organisms consist of at least two functional parts:

- A DNA-binding domain that anchors the transcription factor to the DNA in the region of the gene to be expressed; and
- An activating domain that is required for full activity of the transcription factors.

**Figure 2:**
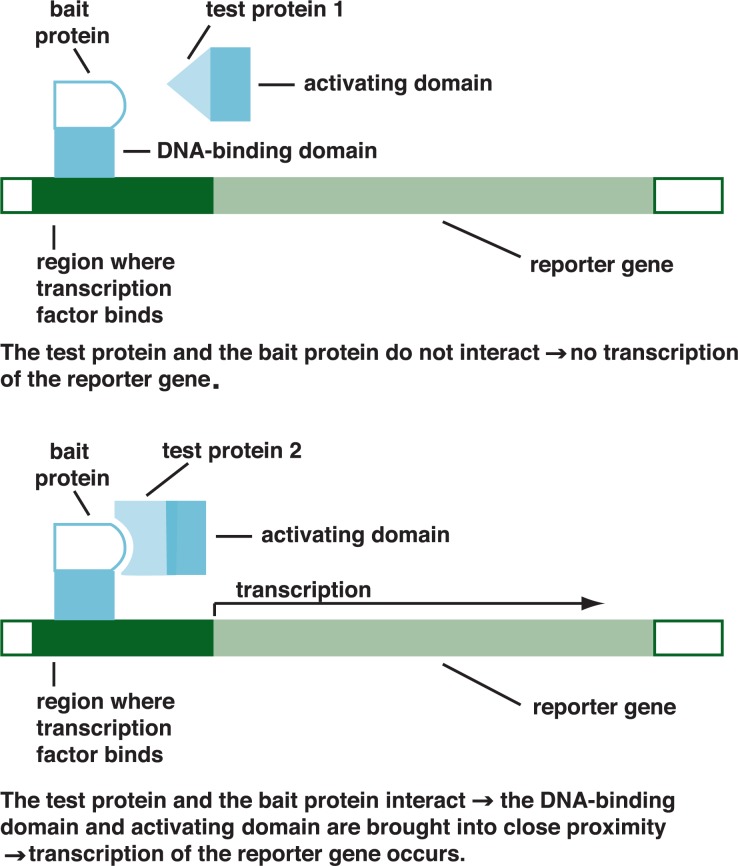
Schematic representation of the yeast two-hybrid screen system. This strategy is based on the fact that certain proteins regulating gene expression in yeast and other higher organisms require two functional parts—a DNA-binding domain and an activating domain—to be fully active and activate the expression of an easily detectable reporter. For this approach, a known “bait” protein is fused to the DNA-binding domain of a yeast transcription factor. The activating domain of that transcription factor is fused to test proteins from a given cell or tissue. The fused test proteins then are mixed with the fused bait. If one of the test proteins interacts with the bait, the DNA-binding and activating domains of the transcription factor are brought together and can stimulate expression of the reporter gene.

Only both domains together can activate expression of a gene. In the yeast two-hybrid approach, a known bait protein is fused through genetic engineering methods to the DNA-binding domain of a yeast transcription factor. The activating domain of that transcription factor, through a series of genetic manipulations, is fused to a whole battery of test proteins from a given cell or tissue. The test proteins fused to the activating domain then are mixed with the bait protein fused to the DNA-binding domain. If one of the test proteins interacts with the bait, the DNA-binding and activating domains of the transcription factor are brought so close together that they become active and stimulate expression of a reporter gene that can easily be detected (figure 2). The test protein that interacted with the bait then can be isolated and studied further.

The yeast two-hybrid technique has been used successfully to identify protein–protein interactions and can be used with many samples simultaneously (i.e., it is amenable to high-throughput applications). However, both the bait and the test proteins (or the genes encoding them) must first be obtained from the original cell (e.g., a human cell) and be manipulated repeatedly through genetic engineering to fuse them to the two domains of the yeast transcription factor. As a result, any observed interactions may be attributed to the artificial conditions of this test system and may have no relevance in the original cell. Therefore, the relevance of the observed interactions must be confirmed using other strategies.

#### Affinity Chromatography

**Figure 3:**
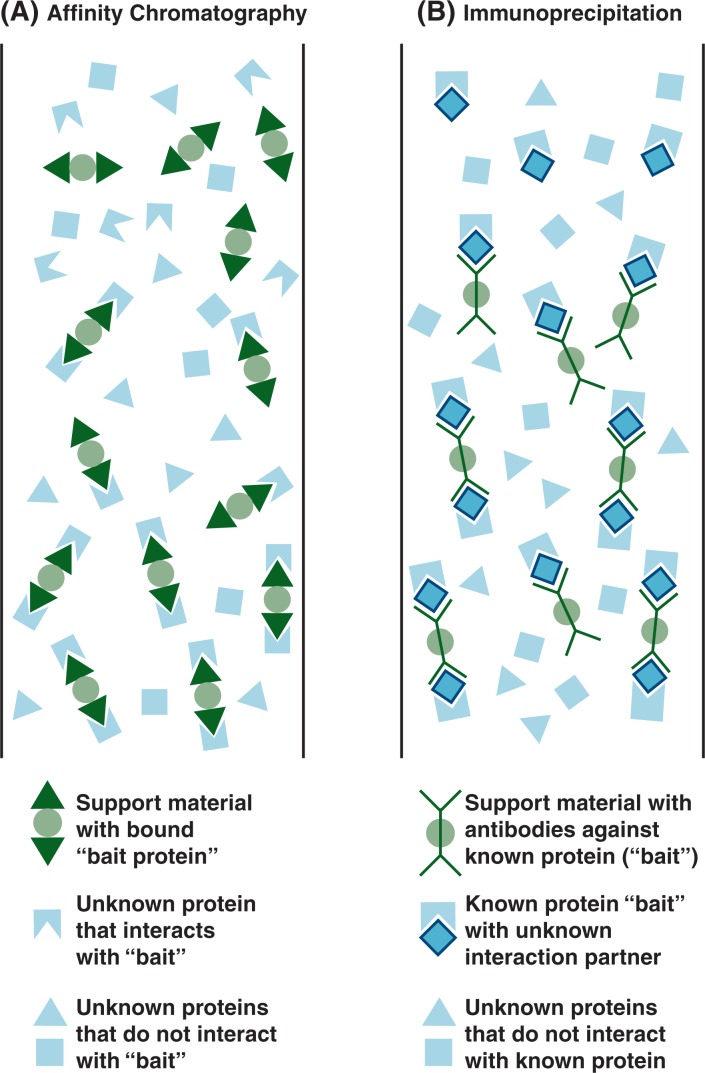
Schematic representation of the principles of affinity chromatography and immunoprecipitation. **A)** For affinity chromatography, a protein known or suspected to be involved in a protein complex (i.e., the “bait”) is attached to a solid support material to generate a stationary phase. Then, a protein extract containing potential interacting proteins is passed through this stationary phase so that complexes between the known protein and other proteins in the extract can form. By passing specific buffer solutions through the solid phase, only proteins involved in specific interactions remain and can be retrieved for further analysis. **B)** During immunoprecipitation, immune molecules called antibodies that interact only with a specific protein are used to pull their known target molecule out of a protein extract and with it any other proteins with which it interacts. If researchers have identified a protein that participates in a protein complex, they can produce antibodies that specifically recognize this “bait” and attach them to a solid support material. When a protein extract containing the bait and any proteins interacting with it is passed through the support, both the bait and the proteins it interacts with stick to the antibodies. Subsequently, the retained material can be retrieved and studied further.

For affinity chromatography approaches, a protein known or suspected to be involved in a protein complex first is attached to a solid support material to generate a stationary phase that, for example, can be loaded into a glass column. Then, a protein extract containing potential interacting proteins is passed through this stationary phase, allowing the complexes between the known protein and other proteins in the extract to form (figure 3A). By passing specific buffer solutions through the columns, any proteins that do not interact specifically with the protein being studied are washed off and only the proteins involved in specific interactions remain. These then can be extracted and analyzed further in subsequent steps, such as MS analyses. The main advantage of the affinity chromatography approach over the classical yeast two-hybrid approach is that the interactions are identified in viable cells or cell extracts and, hence, do not require extensive validation (Aebersold and Mann 2003).

#### Immunoprecipitation

A third approach to studying protein–protein interactions utilizes immune molecules called antibodies to pull a known target molecule out of a protein extract and with it any other proteins with which it interacts. Antibodies are molecules naturally produced by the body that recognize foreign or harmful molecules which have entered the body, bind to these molecules, and mark them for destruction by the body’s immune system. Antibodies that specifically interact with almost any given protein also can be produced in laboratory animals, such as mice, rats, or rabbits. Thus, if researchers have identified a protein that participates in a protein complex, they can produce antibodies that specifically recognize this protein, attach them to a solid support material, and load them onto a chromatography column. Then a protein extract containing the bait protein of interest and any proteins interacting with it is passed through the column. The bait protein—and with it any proteins with which it interacts—sticks to the antibodies and is not washed off the column (figure 3B). Subsequently, the retained, or precipitated, material can be retrieved from the column and studied further. Alternatively, the bait protein can be tagged with a standard chemical group and then immunoprecipitated—together with its interaction partners—by an antibody that specifically recognizes the tagging chemical group (Pandey and Mann 2000). This eliminates the need to generate new antibodies against each new protein to be studied.

Unknown proteins isolated by either of these approaches can be analyzed further by MS to attempt identification. The newly identified proteins then can, in turn, be used to generate antibodies and trap additional interacting proteins, so that eventually an entire network of interacting proteins can be identified.

Immunoprecipitation is a powerful tool to investigate protein–protein interactions and study, for example, the effects of alcohol on certain proteins and protein complexes and on the proteome of certain alcohol-sensitive cells. It enables researchers to isolate the protein of interest and associated protein complex from extracts obtained from the appropriate tissues rather than having to rely on model systems that may lead to incorrect conclusions. However, immunoprecipitation brings some challenges, including the following:

- The antibodies used must bind specifically and tightly to the protein of interest in order to avoid precipitating noninteracting proteins in the sample and to precipitate enough of the protein complexes of interest for further analysis.
- For some proteins it is difficult or impossible to obtain antibodies with these characteristics. In these cases, the tagging approach described above may be helpful.
- The support material to which the antibody is attached often can interact directly with proteins in the extracts studied, resulting in increased “background noise.” This nonspecific binding of proteins to the support material remains one of the main problems in identifying protein complexes using interaction proteomics, and researchers are exploring numerous strategies to minimize its impact. For example, to determine whether an observed interaction is really genuine or just results from the experimental conditions, one can perform a reverse immunoprecipitation (Maiya et al. 2006). This means that if in the original experiment an antibody against protein X was used to precipitate protein X and its novel interaction partner Y, one can then generate an antibody against protein Y to see if it also results in the precipitation not only of protein Y but also of protein X. Only if both proteins always are precipitated together is it likely that they form a complex in the cell.

### Identification of Membrane-Bound Proteins

Approximately 20 to 30 percent of the entire proteome is made up of proteins that are bound to the membranes that surround the cells or form internal structures inside the cells (Stevens and Arkin 2000; Wallin and von Heijne 1998). To date, however, most proteomic studies have focused on soluble proteins; accordingly, information about membrane proteins remains limited. This discrepancy is even more problematic as membrane proteins make up the majority of all targets for pharmaceutical drugs.

Several difficulties are associated with the application of proteomic approaches to the study of membrane proteins. For example, because they normally are bound to membranes, these proteins are not soluble in the buffer solutions typically used for protein digestion, gel electrophoresis, or MS analyses. However, certain modifications to commonly used methodologies can allow application of proteomics to the study of membrane proteins (for a review, see Wu and Yates 2003). For instance, with gel-based methods involving protein separation and subsequent identification using MS, the way in which the proteins are prepared can be modified so that the proteins become more soluble. Also, membrane proteins become insoluble if the buffer in which they are analyzed approaches their isoelectric point; therefore, membrane proteins should be separated on one-dimensional gels instead of two-dimensional gels to eliminate separation based on isoelectric point. However, this approach obviously reduces the resolution of the separated proteins, making identification by MS more difficult.

Another problem with analyzing membrane proteins using proteomic approaches is that, as described earlier, the separated proteins must be treated with trypsin in the gel before they are analyzed by MS. However, either some areas of membrane proteins do not contain trypsin cleavage sites or these sites are not readily accessible to the proteases. To circumvent this problem and improve the fragmentation of membrane proteins, trypsin treatment can be coupled with other protease or chemical digestion methods. Using such a modified solubilization method followed by MudPIT analysis, Washburn and colleagues (2001) performed the first large-scale shotgun proteomic analysis of membrane proteins. These investigators identified 131 integral membrane proteins in yeast.

### Combination of Genomic and Proteomic Approaches

To further evaluate the biological significance of proteomic findings, they can be compared with genomic observations. For example, if shotgun proteomic experiments have identified proteins whose abundance changes in response to alcohol, one can try to analyze whether these changes in expression also are detectable at the mRNA level. Similarly, if several interacting proteins in a protein complex have been identified, one can analyze whether the corresponding genes also are regulated in a coordinated fashion. Most of these analyses can be done using computer data bases (i.e., in silico). Large databases are available that provide gene expression data (e.g., mRNA abundance values) for thousands of genes in a variety of tissues. In many cases, the mRNA corresponding to a specific protein can be identified based on the amino acid sequence of the protein, and any changes in abundance at the protein level can be compared with changes at the mRNA level.

## Recent Results of Proteomic Research in the Alcohol Field

Over the past few years, researchers increasingly have begun to use proteomic analyses to investigate alcohol’s effects on the body. Some investigations have focused on the proteome of tissues known to be affected by alcohol, such as the liver and brain. Most of these studies have used shotgun proteomics to establish a broad picture of how alcohol exerts its effect at the proteome level in these tissues. In addition, shotgun proteomic approaches have been used to search for biomarkers of alcoholism. Other investigators have focused on specific cellular components that are targets of alcohol’s actions, such as molecules that interact with neurotransmitters (i.e., neurotransmitter transporters or receptors). These studies have used interaction proteomics to determine which proteins are present in such protein complexes in order to gain insight into the processes in nerve cell transmission. Finally, investigators have begun to compare the data obtained in proteomic studies with the findings of genomic analyses for a more complete understanding of alcohol’s effect on the body. The following sections summarize the findings of some of these studies.

### Results of Shotgun Proteomic Studies

#### Identification of Brain Proteins Affected by Alcohol

**Figure 4:**
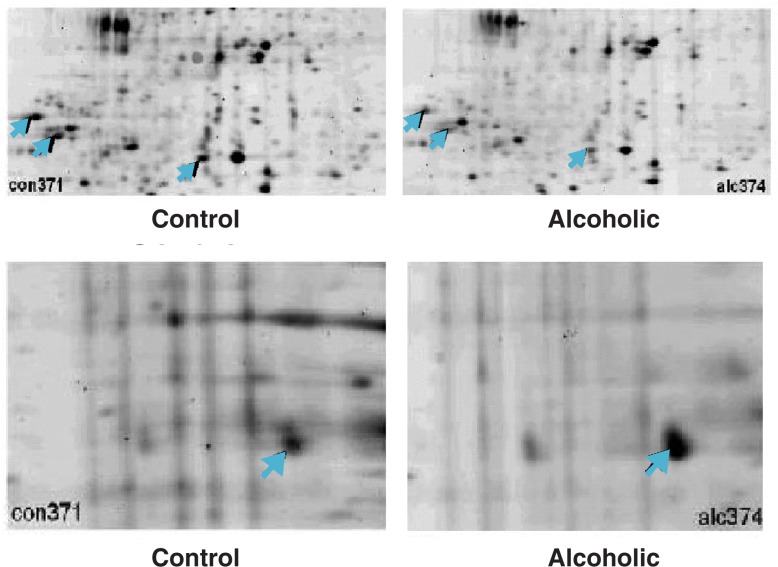
Examples of proteins whose expression is reduced in alcoholics compared with control subjects (upper panel) or enhanced in alcoholics compared with control subjects (lower panel).

Chronic alcohol exposure leads to a wide range of changes in brain function. On the one hand, alcohol damages neurons in various brain regions, leading to cognitive impairment and other abnormalities in brain function in alcoholics. On the other hand, the brain adapts to the constant presence of alcohol, trying to counteract alcohol’s harmful effects. (This adaptation leads to withdrawal symptoms if alcohol levels suddenly drop.) Both alcohol’s damaging effect on the brain and the body’s adaptive response may be mediated, at least in part, by altered protein expression. To evaluate this assumption, Lewohl and colleagues (2004) studied the proteome of one brain region affected by alcohol—the superior frontal cortex (SFC)—of long-term alcoholics and healthy control subjects, using autopsy samples. Using 2-DE, the investigators compared proteins from the healthy and alcoholic subjects for differential expression (i.e., differences in abundance of a given protein of two-fold or more between the two groups). This analysis detected 182 proteins with differential expression, including 139 proteins that were less abundant in alcoholics, 35 proteins that were more abundant in alcoholics, and 8 proteins that were found only in alcoholics or nonalcoholics (see figure 4). However, these analyses did not yet provide any information on the nature and function of these proteins. By subsequently using MALDI-MS and tandem MS, the investigators were able to identify 63 of the 182 proteins; these fell into several groups based on their physiological functions. The researchers concluded that proteomic studies can be conducted on autopsy samples to identify candidate proteins that are affected by long-term alcohol use and whose exact roles can be analyzed further.

In a similar study, Alexander-Kaufman and colleagues (2006) used autopsy samples to compare the proteome of a region located at the front of the brain that is composed mainly of nerve cell extensions (i.e., prefrontal white matter) and which has been shown to be particularly susceptible to alcohol-induced brain damage and shrinkage. The researchers studied tissue samples obtained from alcoholics with and without cirrhosis, one abstinent alcoholic, and nonalcoholics. Using 2-DE of the protein samples researchers detected differences in the expression of 60 proteins, 40 of which showed decreased expression in alcoholics compared with nonalcoholics. Additional MALDI-TOF MS analyses allowed identification of 18 proteins. These included some enzymes important for energy production in the cell, as well as some proteins that previously have been implicated in alcohol-related disorders and brain damage.

Although these studies suggest that chronic alcohol consumption directly alters the levels of a number of important brain proteins, it should be emphasized that such changes also may be an indirect result of other concomitant conditions, such as liver disease. For example, a recent study has shown that brain gene expression changes are greater in alcoholics with cirrhosis than in those without cirrhosis, suggesting that such changes likely contribute to the more severe brain dysfunction in individuals with liver disease (Liu et al. 2007).

Some researchers have applied the shotgun proteomics approach to an animal model of alcohol consumption, comparing the proteome of relevant brain regions from two strains of rats that were bred to be alcohol-preferring (P rats) or nonpreferring (NP rats) (see Neuhold et al. 2004). Using 2-DE and MALDI-TOF MS, the researchers identified 70 proteins whose expression differed significantly between the two rat strains, with the largest differences found for several proteins involved in signaling pathways. Moreover, protein expression generally was lower in the P rats than in the NP rats. Thus, a common theme emerges from this study as well as from the studies by Lewohl and colleagues (2004) and Alexander-Kaufman and colleagues (2006), namely that chronic alcohol use appears to reduce expression of the majority of proteins studied. More recently, a similar study investigated the effect of chronic alcohol drinking in specific regions of the limbic system (i.e., nucleus accumbens and amygdala) in inbred alcohol-preferring (iP) rats (Bell et al. 2006). The investigators identified numerous altered proteins that could be grouped into functional categories, including chaperones, cytoskeleton, intracellular communication, membrane transport, metabolism, energy production, and neurotransmission.

#### Search for Biomarkers

Another area of research where proteomic approaches are being used is the search for biomarkers of alcoholism—proteins that differ in abundance between alcoholics and nonalcoholics and which easily can be measured to determine whether a person has been drinking alcohol recently or is an alcoholic. Kasinathan and colleagues (2004) have used a proteomic approach to search for urinary biomarkers of alcohol intake, which could be useful for monitoring alcoholics during treatment and for identifying people who are at risk for alcoholism. Using 2-DE, the investigators analyzed the proteome of the urine of alcohol-treated and control rats. This analysis revealed several proteins that were present only in the urine of alcohol-treated animals but not in the urine of control animals. Subsequent tandem MS analyses identified one of these proteins as an enzyme called transferrin.^2^ Further analyses need to determine whether these findings from rats also apply to humans and whether transferrin can indeed serve as a reliable biomarker for alcoholism.

In another study to identify potential biomarkers for alcoholism, Freeman and colleagues (2006) analyzed serum proteins in cynomolgus monkeys who did or did not self-administer large quantities of alcohol, looking for differences between the two groups. Using an MS technique called surface-enhanced laser desorption ionization/time of flight (SELDI-TOF), the researchers identified two proteins that were differentially expressed in the two groups. These were identified as apolipoprotein AI and apolipoprotein AII, respectively. Both of these are components of high-density lipoprotein (HDL), commonly known as “good” cholesterol. Additional analyses found that apolipoprotein AII levels were consistently elevated in alcohol-consuming animals, whereas apolipoprotein AI levels were increased only in some animals, consistent with previously published data on human subjects (see Neuhold et al. 2004). This study demonstrates that non-human primates can serve as a model for identifying biomarkers in alcohol research. This is an important discovery, because studies involving human subjects are associated with many disadvantages, such as inconsistent self-reporting of alcohol intake, variations in diet, and other individual differences between the subjects.

### Results of Interaction Proteomic Studies

Protein–protein interactions are involved in many physiological processes, including nerve signal transmission in the brain. For example, it has been known for some time that release of neurotransmitter molecules that transmit the nerve signal from the emitting neuron to the receiving neuron involves regulated protein–protein interactions (Brodin et al. 2000). Moreover, growing evidence indicates that proteins located in the synaptic membrane that allow passage of ions into and out of the cell or that transport neurotransmitters back into the cell also are regulated by complex protein interactions (Deken et al. 2000; Garner et al. 2000; Maiya and Mayfield 2004; Muth et al. 1998; Staub and Rotin 1997; Sung et al. 2005). Therefore, identification and characterization of the protein–protein interactions is central to understanding the processes that occur during the transmission of nerve signals at the synapse and during the changes in brain cell functioning that occur during the brain’s adaptation to the presence of alcohol. However, few of the accessory proteins that interact with synaptic proteins have been identified to date. Thus, the characterization of protein complexes that are altered by alcohol will open new and promising opportunities for better understanding the cellular mechanisms involved in alcohol addiction as well as the regulation of protein function.

As one step in this direction, Maiya and colleagues (2006) attempted to define the dopamine transporter (DAT) proteome. Cells using dopamine as a neurotransmitter (i.e., dopaminergic neurons) are one of the targets of alcohol action in the brain. When a nerve signal is transmitted, dopamine is released from the signal-emitting neuron into a small space (i.e., the synaptic cleft) separating it from the signal-receiving neuron. To end signal transmission, the dopamine must be transported back into the signal-emitting neuron. This transport is achieved by the DAT. Its activity is regulated by multiple signaling mechanisms, at least some of which are likely to involve protein–protein interactions. To understand these regulatory mechanisms (and alcohol’s possible impact on them), it is important to identify the proteins that interact with the DAT. To this end, Maiya and colleagues (2006) used immunoprecipitation to obtain the DAT–protein complexes, one-dimensional gel electrophoresis to separate the precipitated proteins, and MS to analyze individual proteins isolated from the gel. These analyses demonstrated that the dopamine transporter was associated with at least 20 proteins, some of which could be identified with at least some certainty by MS. These included the following:

- Signaling proteins;
- Trafficking proteins that are involved in the transport of proteins and other molecules within and between cells;
- Cell adhesion molecules that allow cells to adhere to each other;
- Ion channels that allow the passage of ions into and out of cells;
- Cytoskeletal proteins that help give cells their three-dimensional structure; and
- Metabolic enzymes.

Although the general functions of these proteins are, for the most part, known, the exact nature of their interactions with the DAT and their specific functions in the DAT complex still need to be confirmed. Future analyses also must determine how alcohol exposure and alcohol dependence influence these interactions.

Similar studies have been conducted for other neurotransmitter systems that also are known to be affected by alcohol, such as the one involving the neurotransmitter glutamate. For example, Husi and colleagues (2000) studied the protein complex making up one of the receptors to which glutamate binds on a signal-receiving neuron, known as the *N*-methyl-d-aspartate (NMDA) receptor. Using interaction proteomic approaches, the researchers determined that the NMDA receptor complex comprised 77 different proteins with a diverse range of functions, such as binding glutamate and initiating intracellular signaling processes that modify the cell’s activity.

### Results of Combined Genomic and Proteomic Approaches

In addition to gathering proteomic data, alcohol researchers also are beginning to combine those data with others obtained in genomic analyses. For example, in their analysis of the DAT proteome, Maiya and colleagues (2006) sought not only to identify proteins that interact with the DAT but also to evaluate the biological significance of these proteins as a group. This was accomplished by determining the expression levels of the genes encoding these proteins using computerized searches of existing databases on gene expression—an approach known as in silico analysis. This analysis was based on previous findings that the expression of genes encoding interacting proteins often is regulated in a coordinated fashion. The analysis found that the correlation between the expression levels of the genes encoding the various interacting proteins was greater than would be predicted by chance alone, suggesting common regulatory mechanisms. In particular, the expression of nine genes was highly correlated, further suggesting common regulatory mechanisms for these genes. These findings support the notion that the interaction of these proteins does indeed have biological significance and does not result solely from the experimental conditions (i.e., is not an artifact).

An equally comprehensive strategy was used by other investigators who sought to determine factors that contribute to anxiety and alcoholism, including signaling pathways that affect the NMDA receptor (see Sikela et al. 2006). These researchers studied mice carrying extra copies of a gene (and therefore producing excess amounts of the corresponding protein) that is associated with an increased risk of anxiety. These animals were compared with mice lacking the extra gene using three approaches:

- A type of genetic analysis called quantitative trait locus (QTL) analysis was used to identify regions of the mouse chromosomes that influence the risk of anxiety.
- Microarray analysis was used to measure the mRNA levels of all expressed genes, yielding a list of genes that were differentially expressed between the two mouse strains. Of these genes, eight were found to be located in the chromosomal regions (QTLs) that had been identified as being associated with the risk of anxiety.
- Shotgun proteomics—specifically MudPIT analyses—of synaptosomes (isolated synapses) from both groups of animals identified several proteins that differed in abundance between the two mouse strains. Five of these proteins were encoded by genes that earlier had been found to be located in the QTL for anxiety.

These examples demonstrate how genomic and proteomic studies can complement each other and how a combination of approaches can help researchers gain a more complete understanding of complex processes in the brain and other organs.

## Conclusions and Future Directions

Proteomic studies increasingly are being used in alcohol research. Advances in the proteomics field have allowed researchers to apply novel techniques in their efforts to elucidate alcohol’s effects on a variety of tissues and to identify the mechanisms by which alcohol can regulate protein function and/or alter the interactions among associated proteins. Most of these studies to date have compared the effects that alcohol has on protein expression in various tissues or cells; these studies typically have used shotgun proteomic approaches. Other investigators are beginning to study protein complexes that may be impacted by alcohol, such as proteins participating in various neurotransmitter systems. However, compared with studies addressing alcohol’s effects on single neurotransmitter receptors and transporters, relatively few studies have focused on multiple interacting proteins as potential sites of action. Changes in one component of a protein complex, however, may impact a range of protein characteristics (e.g., its stability or transport in the cell) that can ultimately alter the entire complex’s response to alcohol. Thus, interaction proteomic studies can provide insight into the direct mechanisms through which alcohol exerts its effect on particular proteins and protein complexes.

To date, few proteomic analyses have dealt directly with the effects of alcohol on specific cells of tissues; instead, most analyses have focused more generally on tissues known to be affected by alcohol. These investigations are laying the foundation for future studies exploring the specific effects of alcohol on the identified proteins (or the components of protein complexes) and their functions, which may contribute to understanding alcohol’s mechanisms of action. Moreover, the results of such analyses may help in the development of biomarkers of alcoholism and susceptibility to alcohol-induced tissue damage as well as in the identification of therapeutic targets.

Although much progress has been made in the technologies used in proteomic investigations, researchers still must be aware of the existing limitations. For example, the choice of assay system (e.g., the choice of separation technique) can influence the results. Some gel materials are more suitable for the separation of membrane-bound proteins and others for soluble proteins. Therefore, the results obtained with a sample will differ depending on the gel material used. Another factor that can affect the outcome is the type of sample used—that is, whether investigators use material from one subject or pool material from several subjects. Similarly, the specific material used (i.e., tissue, serum, specific cell type, or subcellular fraction) can play a role. All these differences in experimental designs may be responsible for variability in findings.

The combination of genomic and proteomic approaches, including the use of in silico approaches, has great potential to further increase understanding of the multiple mechanisms through which alcohol affects all organs of the body. Thus, the findings of these studies are essential both for understanding the pathogenesis of alcohol dependence and for identifying therapeutic targets to treat alcoholism and prevent some of the deleterious effects of alcohol.

## Footnotes

1 For a definition of this and other technical terms, see the glossary on p. 84.

2 A modified form of transferrin, called carbohydrate-deficient transferrin, which can be measured using blood tests, already is being used by researchers and clinicians as a marker for chronic alcohol consumption (Miller and Anton 2004).
